# Microwave Assisted Synthesis, Antifungal Activity and DFT Theoretical Study of Some Novel 1,2,4-Triazole Derivatives Containing the 1,2,3-Thiadiazole Moiety

**DOI:** 10.3390/molecules181012725

**Published:** 2013-10-15

**Authors:** Na-Bo Sun, Jian-Qun Fu, Jian-Quan Weng, Jian-Zhong Jin, Cheng-Xia Tan, Xing-Hai Liu

**Affiliations:** 1 College of Biology and Environmental Engineering, Zhejiang Shuren University, Hangzhou 310015, Zhejiang, China; E-Mails: nabosun@gmail.com (N.-B.S.); fujianqunshuren@126.com (J.-Q.F.); jinjianzhongshuren@gmail.com (J.-Z.J.); 2 College of Chemical Engineering and Materials Science, Zhejiang University of Technology, Hangzhou 310014, Zhejiang, China; E-Mails: jqweng@zjut.edu.cn (J.-Q.W.); tangchengxia@zjut.edu.cn (C.-X.T.)

**Keywords:** 1,2,4-triazole, 1,2,3-thiadiazole, synthesis, fungicidal activity, thioether, theoretical calculations

## Abstract

In order to investigate the biological activity of 1,2,4-triazole compounds, seventeen novel 1,2,4-triazole derivatives containing 1,2,3-thiadiazole moieties were synthesized by multi-step reactions under microwave assisted conditions. The structures were characterized by ^1^H-NMR, ^13^C-NMR, MS and elemental analyses. The target compounds were evaluated for their *in vivo* fungicidal activities against *Corynespora cassiicola*, *Pseudomonas syringae* pv. *Lachrymans*, and *Pseudoperonospora cubensis*, and the results indicated that some of the title compounds displayed good fungicidal activities. Theoretical calculations on the title compounds were carried out at the B3LYP/6-31G (d,p). level. The full geometry optimization was carried out using the 6-31G(d,p) basis set, and the frontier orbital energy, atomic net charges were discussed, and the structure-activity relationships were also studied.

## 1. Introduction

**Figure 1:**
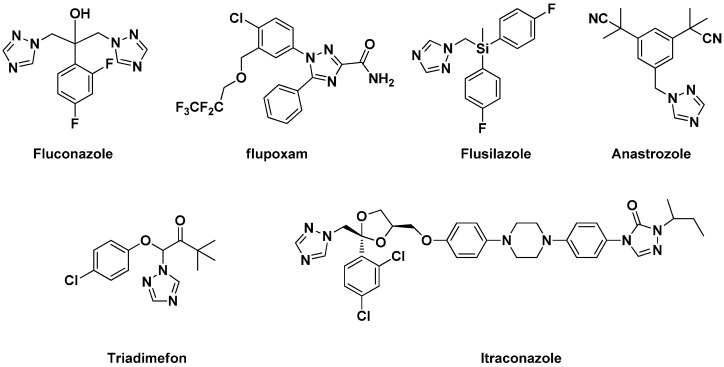
Commercial drugs and pesticides containing 1,2,4-triazole groups.

In recent years, heterocyclic compounds had been receiving considerable attention due to their pharmacological and pesticidal importance [1,2,3,4,5,6,7,8,9]. Nitrogen-containing heterocycles exhibit excellent biological activities, especially 1,2,4-triazoles and 1,2,3-thiadiazoles. 1,2,4-Triazole rings are typically planar 6*π*-electron aromatic systems, featuring an extensive chemistry [10,11]. 1,2,4-Triazole and its derivatives represent one of the most biologically active classes of compounds, displaying a diversity bioactivities in the medicinal and agrochemical field, including anti-inflammatory [12,13], antifungal [14,15], herbicidal [16], antimicrobial [16,17], antiparasitic [18], cytostatic [19], and brassinosteroid biosynthesis inhibitory activities [20]. Some such compounds had been developed as commercial fungicides or herbicides (Figure 1), such as triadimefon, triadimenol, flusilazole, flupoxam and so on.

**Figure 2:**
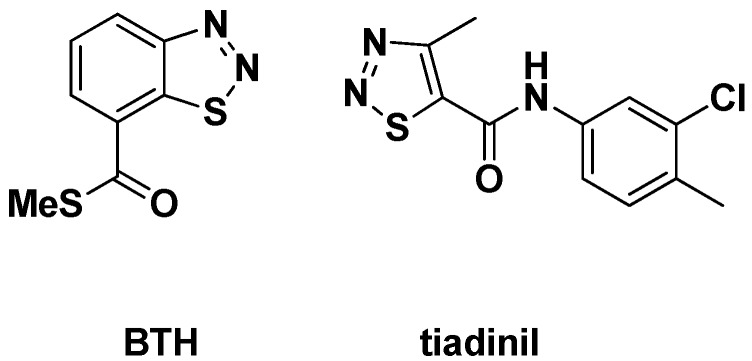
Some commercial pesticides containing 1,2,3-thiadiazoles.

1,2,3-Thiadiazoles have also been claimed to have beneficial agricultural applications [21,22], because they exhibit wide spectrum biological activities, such as antiviral [23], herbicidal [24,25], antiamoebic [26] and insecticidal [27] activities. For example, after plant inducers such as tiadinil (TDL) and acibenzolar-S-methyl (BTH) (Figure 2) were discovered, 1,2,3-thiadiazole pesticides have become one of the focuses for developing agrochemicals in academia and industry.

There are many reports about each of the two heterocycles, but the combination of 1,2,3-thiadiazole ring with 1,2,4-triazole ring in one molecule is seldom reported either in chemistry or biological activity studies. In view of these facts mentioned above, and also as a part of our work [28] on the synthesis of bioactive lead compounds, the title compounds were designed by introducing the 1,2,3-thiadiazole pharmacophore into a 1,2,4-triazole scaffold. Seventeen novel 1,2,4-triazole derivatives were synthesized and characterized by ^1^H-NMR, ^13^C-NMR, MS and elemental analysis. The antifungal activities of these compounds were tested *in vivo.*

## 2. Results and Discussion

### 2.1. Chemistry

**Scheme 1:**
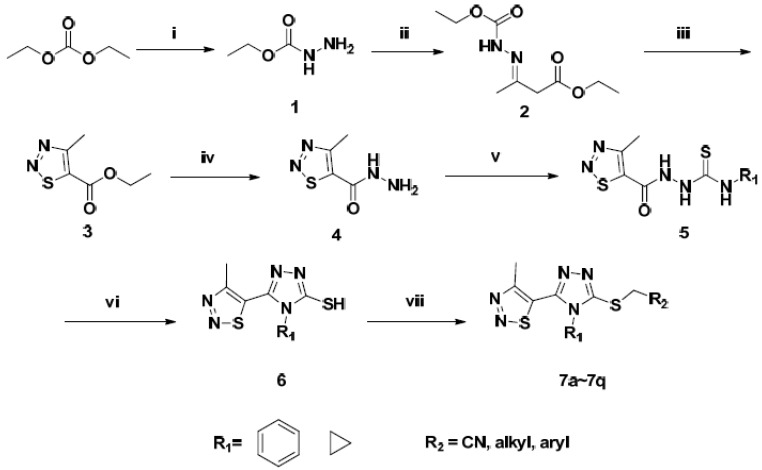
The synthetic route of title compounds.

The synthesis procedures for title compounds are shown in Scheme 1. The intermediates **1**~**6** were synthesized according our previous method [25]. The intermediate **1** was easily synthesized from diethyl carbonate and 85% hydrazine hydrate at room temperature. The method of synthesis of the Schiff base **2** was conventional. The key intermediate ethyl 4-methyl-1,2,3-thiadiazole-5-carboxylate (**3**) was synthesized using the Hurd-Mori method. Then intermediate **3** is reacted with 85% hydrazine hydrate to give intermediate **4**. The intermediate **5** can be obtained from the intermediate **4** and a substituted isothiocyanic ester. The intermediate **5** was easily cyclized to give intermediate **6** under alkaline conditions, such as NaOH. The intermediate **6** is reacted with substituted benzyl chlorides or alkyl chlorides to afford compounds **7**. The microwave irradiation assisted synthesis and conventional method were also employed in these experiments. The NaOH/DMF/H_2_O system was applied under microwave irradiation. The best reaction conditions were 90 °C for 15 min under microwave irradiation. The yield is higher than that of the conventional method, and the reaction time is also shorter (Table 1).

*Reagents and conditions*: (i) NH_2_NH_2_ H_2_O, EtOH, reflux; (ii) ethyl 3-oxobutanoate, EtOH, room temperature; (iii) SOCl_2_, room temperature; (iv) NH_2_NH_2_ H_2_O, EtOH, reflux; (v) R_1_NCS, EtOH, reflux; (vi) a. NaOH, reflux; b.HCl; (vii) R_2_CH_2_Cl, K_2_CO_3_, DMF, MW.

**Table 1:** Comparison of yields of **7b** with or without microwave irradiation.

No.	Method	Time	Temperature/°C	Yield/%
**7b**	No-MW	24 h	r.t.	78
**7b**	MW	10 min	90	80
**7b**	MW	15 min	90	85
**7b**	MW	20 min	90	83

The signal of the CH_2_ protons of the thioether neighboring the triazole ring was observed at *δ* 4.43~4.63 ppm, respectively. The chemical shifts around 3.0 ppm are from the methyl of the 1,2,3-thiadiazole ring. The ESI-MS spectra showed the *m/z* of the molecular ions, according with their molecular formulas. The elemental analysis results were in accord with the calculated results.

### 2.2. Fungicidal Activities and Structure-Activity Relationship (SAR)

The *in vivo* fungicidal results of the title compounds against *Corynespora cassiicola (CC)*, *Pseudomonas syringae* pv. *Lachrymans (PS)* and *Pseudoperonospora cubensis (PC)* are listed in Table 2, where iprodione, validamycin and topsin-M were used as controls. As shown in Table 2, lots of the title compounds showed good control efficacy against *Corynespora cassiicola* at a concentration of 500 μg/mL. When the R_1_ is a phenyl group, only compound **7e** exhibited moderate activity (46.9%) against *Corynespora cassiicola*. Notably, compounds **7b**, **7c**, **7d** and **7e** displayed excellent activity (>80%) against *Corynespora cassiicola*, especially compound **7b** which exhibited higher activity than the control. Also the activity of benzene substituted derivatives is higher than that of cyclopropyl substituted ones. Compounds **7b**, **7c**, **7d**, **7j**, **7k**, **7l**, **7m** exhibited fair inhibition effects against *Pseudomonas syringae* pv. *Lachrymans*, and the fungicidal activities (control efficacy of 52%–79%) were higher than those of iprodione, validamycin and topsin-M. All of them were less effective than the control validamycin. Compound **7e** (71.03%) and **7g** (68.79%) showed about 70% control effect against *Pseudoperonospora cubensis*, at the same level as iprodione, validamycin and topsin-M. In general, the *in vivo* fungicidal activity of compounds **7 ** indicated that the substituent changes affect the activity.

In order to further research the structure–activity relationships of the title compounds, some compounds (**7o**, **7p**, **7q**) containing cyclopropyl groups synthesized in our other paper [29] were used for comparison. From the data in Table 2, for *Corynespora cassiicola*, the compounds containing phenyl groups exhibited higher activity than those with cyclopropyl groups, but for *Pseudoperonospora cubensis*, the results are the opposite, and the compounds containing cyclopropyl groups displayed higher activity than that of phenyl group-containing compounds. For *Pseudomonas syringae* pv. *Lachrymans*, the structure–activity relationships are not obvious. Further biological evaluation of all compounds is in progress. The mode of action of title compounds will be explored by molecular modeling.

**Table 2:** The antifungal activity of title compounds *in vivo*.

No.	R_1_	R_2_	*CC*	*PS*	*PC*
**7a**	Ph	2,4-Cl_2_Ph	72.97	−1.71	50.34
**7b**	Ph	Ph	93.19	60.67	54.51
**7c**	Ph	4-F Ph	82.77	55.08	44.40
**7d**	Ph	3-Cl Ph	82.30	53.92	39.15
**7e**	Ph	3-F Ph	46.90	36.71	71.03
**7f**	Ph	2-F Ph	75.93	17.59	52.88
**7g**	Ph	4-MePh	84.96	47.16	68.79
**7h**	Ph	4-Et Ph	70.17	11.49	19.65
**7i**	Ph	CH_3_CH_2_	75.22	48.20	42.25
**7j**	Ph	2-Cl Ph	75.15	61.48	28.64
**7k**	Ph	CN	75.75	67.06	26.15
**7l**	Ph	4-Bu Ph	59.92	61.00	49.38
**7m**	Ph	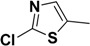	70.64	63.09	18.28
**7n**	Ph	3,4-Cl_2_Ph	71.07	79.76	39.35
**7o**	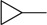	3-CN Ph	28.99	24.41	69.17
**7p**	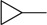	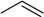	21.38	6.47	71.07
**7q**	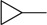	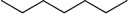	54.03	30.17	81.62
iprodione			53.52	38.49	55.92
validamycin			78.20	−11.42	56.31
topsin-M			78.75	54.15	68.71

(a) *CC*: *Corynespora cassiicola*; *PS*: *Pseudomonas syringae* pv. *Lachrymans*; *PC*: *Pseudoperonospora cubensis*; (b) The test concentration of antifungal activity is at 500 mg/mL.

### 2.3. Molecular Total Energies and Frontier Orbital Energy Analysis

Molecular total energy and frontier orbital energy levels are listed in Table 3. Energy gap between HOMO and LUMO was calculated by B3LYP.

**Table 3:** Total energy, frontier orbital energy.

	DFT
*E*_total_/Hartree ^b^	−1765.0575
*E*_HOMO_/Hartree	−0.221
*E*_LUMO_/Hartree	−0.069
Δ*E*^a^/Hartree	0.152

^a^ Δ*E*= *E*_LUMO_−*E*_HOMO_; ^b^ 1 Hartree = 4.35974417 × 10^−18^ *J* = 27.2113845 eV.

**Figure 3:**
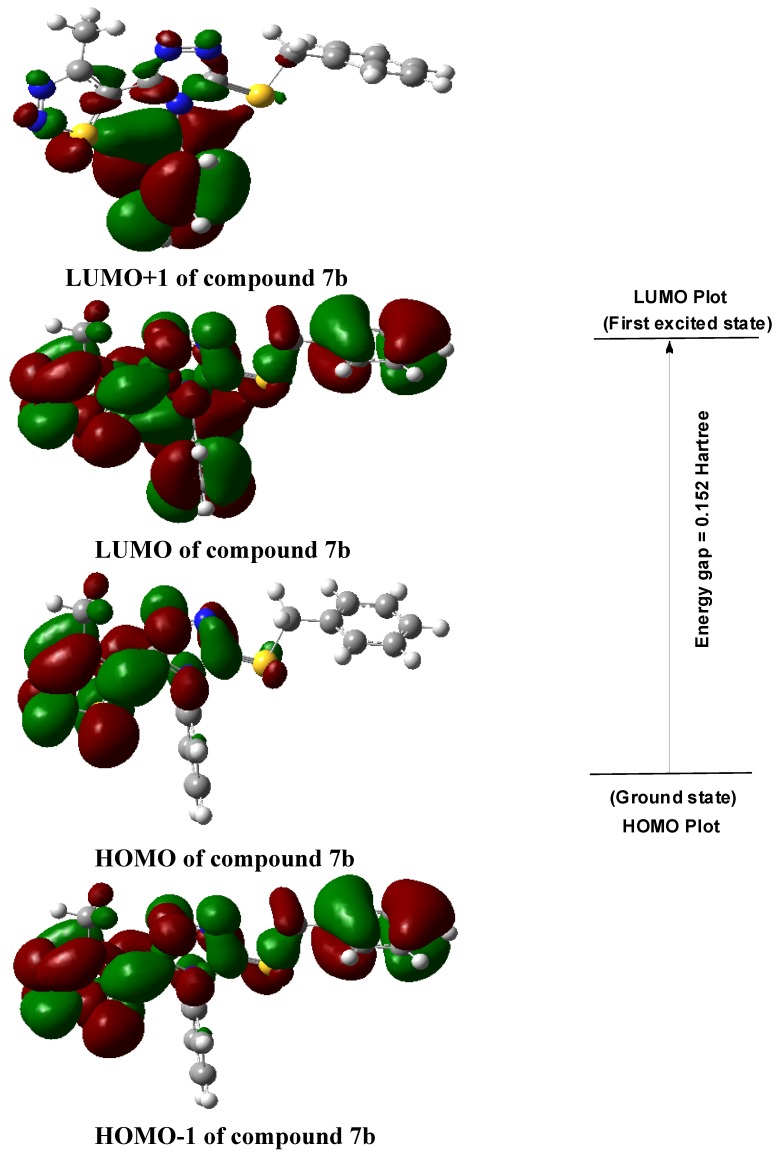
The HOMO and LUMO of compound **7b**.

According to the frontier molecular orbital theory, HOMO and LUMO are the most important factors that affect the bioactivity. HOMO has the priority to provide electrons, while LUMO can accept electrons first [30]. Thus study on the frontier orbital energy can provide useful information about the biological mechanism. Taking the DFT result for example, the geometry of the frame of the title compound is hardly influenced by the introduction of either a 1,2,3-thiadiazole ring, 1,2,4-triazole ring, thioether group or phenyl ring (Figure 3). The HOMO of the title compound is mainly located on the 1,2,3-thiadiazole ring, 1,2,4-triazole ring and thioether group, while, the LUMO of the title compound is located on the 1,2,3-thiadiazole ring, 1,2,4-triazole ring, thioether group and phenyl ring. The fact that the title compound has strong affinity suggests the importance of the frontier molecular orbital in the π-π stacking or hydrophobic interactions. This also implies that the orbital interactions between the title compound and the aromatic ring or some other side of residue chains of receptors is dominated by π-π or hydrophobic interactions among the frontier molecular orbitals.

### 2.4. Mulliken Atomic Charges

Table 4 lists the calculated Mulliken atomic charges except for the H atoms. Taking DFT for example again, three atoms C1, C2, C17, S1 and S2 are the most positively charged ones, which can interact with the negative charged part of the receptor easily. The negative charges are mainly located on atoms N1, N2, N3, N4 and N5, so they can interact easily with the positive part of the receptor. Therefore, we supposed that this compound can combine the amino-acid residues on its surface by the interaction of S2-N4-N5 and S1-C1-N3, which may account in part for the bioactivity.

**Table 4:** Mulliken atomic charges except for H atoms (e).

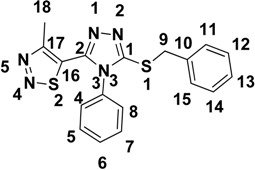	
Atom	Charge
	DFT
C1	0.150691
C2	0.374005
C3	0.208092
C4	−0.011153
C5	−0.003467
C6	−0.000164
C7	−0.000969
C8	0.001182
C9	−0.123948
C10	0.085700
C11	−0.009788
C12	0.010835
C13	0.013612
C14	0.011859
C15	−0.010715
C16	−0.199074
C17	0.267633
C18	0.042000
N1	−0.339205
N2	−0.155032
N3	−0.504897
N4	−0.242781
N5	−0.267556
S1	0.295248
S2	0.407893

## 3. Experimental

### 3.1. Materials and Reagents

All the reagents were of analytical grade or freshly prepared before use. The monitoring of the progress of all reactions and homogeneity of the synthesized compounds were carried out by thin chromatography (TLC), performed on silica gel plates obtained from Qingdao Ocean Chemicals (Qing Dao, China). Melting points were determined using an X-4 apparatus and are uncorrected. ^1^H-NMR and ^13^C-NMR spectra were measured on a Bruker AV 400 or 500 instrument using TMS as an internal standard and CDCl_3_ as the solvent. Mass spectra were recorded on a Thermo Finnigan LCQ Advantage LC/mass detector instrument. Elemental analyses were performed on a Vario EL elemental analyzer. Microwave activation was carried out with a CEM Discover^TM^ focused microwave (2,450 MHz, 300 W).

### 3.2. Therotical Calculations

On the basis of the above structure shown in Scheme 1, compound 7b was selected as the initial structure, and the DFT-B3LYP/6-31G (d,p) [31] methods in the Gaussian 03 package [32] were used to optimize the structure of the title compound. Vibration analysis showed that the optimized structures were in accordance with the minimum points on the potential energy surfaces. All the convergent precisions were the system default values, and all the calculations were carried out on a personal computer.

### 3.3. Chemical Synthesis

#### 3.3.1. Synthesis of Intermediates

##### 3.3.1.1. Preparation of *4-methyl-1,2,3-thiadiazole-5-carbohydrazide* (compounds **1**~**4**)

The synthetic route is shown in Scheme 1. The intermediate 4-methyl-1,2,3-thiadiazole-5-carboxylic acid hydrazide (**2**) was synthesized according to the literature [25]. Carbonic acid diethyl ester (11.8 g, 0.1 mol) and hydrazine hydrate (5.6 mL, 0.095 mol, 85%) were added into a 250 mL round-bottom flask equipped with a condenser. The reaction mixture was heated to 50 °C and stirred for 20 min, and then cooled down to room temperature and further stirred for 30 h. Water, ethanol, and excess carbonic acid diethyl ester were distilled off under reduced pressure. After drying, compound (**2**) (9.88 g, 95% yield) was obtained as white crystals. To a stirred solution of compound 2 (6.36 g, 0.06 mol) in ethanol (16.7 mL), a solution of ethyl acetoacetate (7.8 g, 0.06 mol) in ethanol (3.7 mL) was added at room temperature. Stirring was continued for 6 h. Then, the solvent was removed *in vacuo* and the crude 3-ethoxycarbonyl hydrazonoacetic acid ethyl ester was directly used in the next step without further purification. 3-Ethoxycarbonyl hydrazonoacetic acid ethyl ester (12.8 g, 0.06 mol) was dissolved in dry dichloromethane (25 mL), and thionyl chloride (20 mL) was added to the stirred reaction mixture dropwise at 0 °C for 1 h. Next, the reaction mixture was allowed to stand for 20 h at room temperature. The excess thionyl chloride and dichloromethane were distilled off, and the remaining residue was subjected to fractional distillation under reduced pressure. A slightly yellowish oil of **3** (7.95 g, 77% yield) was obtained. A mixture of 4-methyl-1,2,3-thiadiazole-5-carboxylic acid ethyl ester (1.72 g, 10 mmol) and hydrazine hydrate (12 mmol) in 10 mL of methanol was stirred vigorously for 0.5 h at room temperature, the resulting mixture was then filtered, and the solid was washed with cold methanol. After drying, the solid was recrystallized from methanol to give intermediate (**4**). 

##### 3.3.1.2. *2-(4-Methyl-1,2,3-thiadiazole-5-carbonyl)-N-phenylhydrazinecarbothioamide* (**5**)

A mixture of 4-methyl-1,2,3-thiadiazole-5-carbohydrazide and isothiocyanatobenzene was refluxed for 3 h in ethanol. After cooling down to room temperature, the product obtained was recrystallized from methanol to give **5**.

##### 3.3.1.3. *5-(4-Methyl-1,2,3-thiadiazol-5-yl)-4-phenyl-4H-1,2,4-triazole-3-thiol* (**6**)

A mixture of compound **5** (10 mmol) in aqueous NaOH solution (5 mL, 2N) was refluxed for 4 h. After cooling down to room temperature, HCl aqueous solution (4N) was added to afford a large amount of precipitate. The solid was filtered, dried and recrystallized from methanol to give intermediate **6**.

##### 3.3.1.4. General Procedure for the Preparation of Thioethers **7**

A CEM-designed 10 mL pressure-rated vial was charged with DMF (15 mL), **6** (1.5 g, 5.1 mmol) and K_2_CO_3_ (0.2 g, 5.6 mmol). The mixture was irradiated in a CEM Discover Focused Synthesizer (150 w, 90 °C, 200 psi, 15 minutes). The mixture was cooled to room temperature by passing compressed air through the microwave cavity for 2 minutes. It was poured into cold ice (40 mL) and the precipitate formed was filtered. The crude solid was recrystallized from EtOH to give the title compound **7a**. All the other compounds are synthesized according to the same procedure.

*5-(5-((2,4-Dichlorobenzyl)thio)-4-phenyl-4H-1,2,4-triazol-3-yl)-4-methyl-1,2,3-thiadiazole* (**7a**). White crystals, yield 74%, m.p. 102–103 °C; ^1^H-NMR (CDCl_3_, 400 MHz) d: 3.07 (s, 3H, Het-Me), 4.61 (s, 2H, SCH_2_), 7.14–7.22 (m, 4H, ArH), 7.35–7.40 (m, 2H, ArH), 7.56–7.60 (m, 1H, ArH), 7.64–7.66 (m, 1H, ArH). MS (ESI), *m/z*: 435 (M+1)^+^. Elemental anal. (%), calculated: C, 49.77; H, 3.02; N, 16.12; found: C, 49.98; H, 3.12; N,16.09.

*5-(5-Benzylthio)-4-phenyl-4H-1,2,4-triazol-3-yl)-4-methyl-1,2,3-thiadiazole* (**7b**). White crystals, yield 75%, m.p. 120–121 °C; ^1^H-NMR (CDCl_3_, 400 MHz) d: 3.07 (s, 3H, Het-Me), 4.63 (s, 2H, SCH_2_), 7.08 (d, *J* = 7.99 Hz, 2H, ArH), 7.28–7.32 (m, 3H, ArH), 7.36–7.38 (m, 2H, ArH), 7.54 (t, *J* = 8.00 Hz, 2H, ArH), 7.64 (t, *J* = 7.38 Hz, 1H, ArH). MS (ESI), *m/z*: 366 (M+1)^+^. Elemental anal. (%), calculated: C, 59.15; H, 4.14; N, 19.16; found: C, 58.89; H, 4.43; N,19.32.

*5-(5-((4-Fluorobenzyl)thio)-4-phenyl-4H-1,2,4-triazol-3-yl)-4-methyl-1,2,3-thiadiazole* (**7c**). White crystals, yield 82%, m.p. 88–89 °C; ^1^H-NMR (CDCl_3_, 400 MHz) d: 3.06 (s, 3H, Het-Me), 4.51 (s, 2H, SCH_2_), 6.97 (d, *J* = 8.65 Hz, 2H, ArH), 7.13 (d, *J* = 7.30 Hz, 2H, ArH), 7.36–7.39 (m, 2H, ArH), 7.57 (t, *J* = 8.05 Hz, 2H, ArH), 7.65 (t, *J* = 7.47 Hz, 1H, ArH). MS (ESI), *m/z*: 384 (M+1)^+^. Elemental anal. (%), calculated: C, 56.38; H, 3.68; N, 18.26; found: C, 56.12; H, 3.43; N,18.32. 

*5-(5-((3-Chlorobenzyl)thio)-4-phenyl-4H-1,2,4-triazol-3-yl)-4-methyl-1,2,3-thiadiazole* (**7d**). White crystals, yield 86%, m.p. 101–102 °C; ^1^H-NMR (CDCl_3_, 400 MHz) d: 3.04 (s, 3H, Het-Me), 4.47 (s, 2H, SCH_2_), 7.10 (d, *J* = 7.60 Hz, 2H, ArH), 7.21–7.28 (m, 3H, ArH), 7.36 (s, 1H, ArH), 7.56 (t, *J* = 7.25 Hz, 2H, ArH), 7.65 (t, *J* = 7.18 Hz, 1H, ArH). MS (ESI), *m/z*: 400 (M+1)^+^. Elemental anal. (%), calculated: C, 54.06; H, 3.53; N, 17.51; found: C, 54.33; H, 3.63; N,17.67.

*5-(5-((3-Fluorobenzyl)thio)-4-phenyl-4H-1,2,4-triazol-3-yl)-4-methyl-1,2,3-thiadiazole* (**7e**). White crystals, yield 88%, m.p. 98–99 °C; ^1^H-NMR (CDCl_3_, 400 MHz) d: 3.06 (s, 3H, Het-Me), 4.52 (s, 2H, SCH_2_), 6.97 (t, *J* = 8.25 Hz, 1H, ArH), 7.09–7.24 (m, 4H, ArH), 7.56 (t, *J* = 7.86 Hz, 2H, ArH), 7.63-7.69 (m, 2H, ArH). MS (ESI), *m/z*: 384 (M+1)^+^. Elemental anal. (%), calculated: C, 56.38; H, 3.68; N, 18.26; found: C, 56.75; H, 3.63; N,18.49.

*5-(5-((2-Fluorobenzyl)thio)-4-phenyl-4H-1,2,4-triazol-3-yl)-4-methyl-1,2,3-thiadiazole* (**7f**). White crystals, yield 78%, m.p. 104–105 °C; ^1^H-NMR (CDCl_3_, 400 MHz) d: 3.05 (s, 3H, Het-Me), 4.50 (s, 2H, SCH_2_), 7.05 (d, *J* = 7.70 Hz, 2H, ArH), 7.17–7.32 (m, 4H, ArH), 7.52 (t, *J* = 8.09 Hz, 2H, ArH), 7.61 (t, *J* = 6.97 Hz, 1H, ArH). MS (ESI), *m/z*: 384 (M+1)^+^. Elemental anal. (%), calculated: C, 56.38; H, 3.68; N, 18.26; found: C, 56.13; H, 3.77; N,18.34.

*4-Methyl-5-(5-((4-methylbenzyl)thio)-4-phenyl-4H-1,2,4-triazol-3-yl)-1,2,3-thiadiazole* (**7g**). White crystals, yield 86%, m.p.115–116 °C; ^1^H-NMR (CDCl_3_, 400 MHz) d: 2.33 (s, 3H, Ar-Me), 3.06 (s, 3H, Het-Me), 4.55 (s, 2H, SCH_2_), 7.02 (d, *J* = 7.31 Hz, 2H, ArH), 7.05–7.22 (m, 3H, ArH), 7.29 (d, *J* = 7.45 Hz, 1H, ArH), 7.52 (t, *J* = 7.98 Hz, 2H, ArH), 7.62 (t, *J* = 7.47 Hz, 1H, ArH). MS (ESI), *m/z*: 380 (M+1)^+^. Elemental anal. (%), calculated: C, 60.13; H, 4.52; N, 18.45; found: C, 59.89; H, 4.67; N,18.60. 

*4-Methyl-5-(5-((4-ethylbenzyl)thio)-4-phenyl-4H-1,2,4-triazol-3-yl)-1,2,3-thiadiazole* (**7h**). White crystals, yield 89%, m.p. 135–136 °C; ^1^H-NMR (CDCl_3_, 400 MHz) d: 1.28 (t, 3H, Me), 2.63 (q, 2H, Ar-CH_2_), 3.06 (s, 3H, Het-Me), 4.55 (s, 2H, SCH_2_), 7.06–7.21 (m, 5H, ArH), 7.51–7.62 (m, 4H, ArH). MS (ESI), *m/z*: 394 (M+1)^+^. Elemental anal. (%), calculated: C, 61.04; H, 4.87; N, 17.80; found: C, 60.99; H, 4.97; N,17.56.

*4-Methyl-5-(4-phenyl-5-(propylthio)-4H-1,2,4-triazol-3-yl)-1,2,3-thiadiazole* (**7i**). White crystals, yield 59%, m.p. 106–107°C; ^1^H-NMR (CDCl_3_, 400 MHz) d: 1.03 (t, *J* = 7.37 Hz, 3H, Me), 1.79–1.86 (m, 2H, CH_3_CH_2_), 3.06 (s, 3H, Het-Me), 3.30 (t, *J* = 7.17 Hz, 3H, CH_3_CH_2_CH_2_), 4.55 (s, 2H, SCH_2_), 7.23–7.25 (m, 2H, ArH), 7.58–7.68 (m, 3H, ArH). MS (ESI), *m/z*: 318 (M+1)^+^. Elemental anal. (%), calculated: C, 52.97; H, 4.76; N, 22.06; found: C, 52.76; H, 4.67; N,21.96.

*5-(5-((3-Chlorobenzyl)thio)-4-phenyl-4H-1,2,4-triazol-3-yl)-4-methyl-1,2,3-thiadiazole* (**7j**). White crystals, yield 85%, m.p. 138–139 °C; ^1^H-NMR (CDCl_3_, 400 MHz) d: 3.06 (s, 3H, Het-Me), 4.49 (s, 2H, SCH_2_), 7.10 (d, *J* = 7.16 Hz, 2H, ArH), 7.22–7.25 (m, 1H, ArH), 7.27–7.29 (m, 1H, ArH), 7.37 (s, 1H, ArH), 7.57 (t, *J* = 8.00 Hz, 2H, ArH), 7.66 (d, *J* = 7.58 Hz, 1H, ArH). MS (ESI), *m/z*: 400 (M+1)^+^. Elemental anal. (%), calculated: C, 54.06; H, 3.53; N, 17.51; found: C, 53.89; H, 3.55; N,17.78.

*2-((5-(4-Methyl-1,2,3-thiadiazol-5-yl)-4-phenyl-4H-1,2,4-triazol-3-yl)thio)acetonitrile* (**7k**). White crystals, yield 84%, m p. 118–119 °C; ^1^H-NMR (CDCl_3_, 500 MHz) d: 3.08 (s, 3H, Het-Me), 4.16 (s, 2H, SCH_2_), 7.28 (d, *J* = 7.7 Hz, 1H, ArH), 7.34 (d, *J* = 7.7 Hz, 1H, ArH), 7.63–7.73 (m, 3H, ArH). ^13^C-NMR (CDCl_3_, 125 MHz) d: 14.8, 17.8, 115.1, 127.8, 130.6, 131.7, 132.2, 148.8, 150.7, 160.4. MS (ESI), *m/z*: 315 (M+1)^+^. Elemental anal. (%), calculated: C, 49.66; H, 3.21; N, 26.73; found: C, 49.78; H, 3.11; N,26.55.

*5-(5-((4-(tert-Butyl)benzyl)thio)-4-phenyl-4H-1,2,4-triazol-3-yl)-4-methyl-1,2,3-thiadiazole* (**7l**). White crystals, yield 86%, m.p. 125–126 °C; ^1^H-NMR (CDCl_3_, 500 MHz) d: 1.31 (s, 9H, Bu), 3.07 (s, 3H, Het-Me), 4.52 (s, 2H, SCH_2_), 7.09 (d, *J* = 8.0 Hz, 2H, ArH), 7.29 (d, *J* = 8.5 Hz, 2H, ArH), 7.33 (d, *J* = 8.5 Hz, 2H, ArH), 7.54 (t, *J* = 8.1 Hz, 2H, ArH), 7.63 (d, *J* = 7.5 Hz, 1H, ArH). ^13^C-NMR (CDCl_3_, 125 MHz) d: 14.8, 31.3, 34.6, 36.9, 125.7, 127.9, 128.9, 130.5, 131.5, 132.1, 132.9, 133.7, 147.6, 151.1, 154.3, 159.9. MS (ESI), *m/z*: 423 (M+1)^+^. Elemental anal. (%), calculated: C, 62.68; H, 5.50; N, 16.61; found: C, 62.77; H, 5.55; N,16.45.

*5-(5-(((2-Chlorothiazol-5-yl)methyl)thio)-4-phenyl-4H-1,2,4-triazol-3-yl)-4-methyl-1,2,3-thiadiazole* (**7m**). White crystals, yield 89%, m.p. 129–130 °C; ^1^H-NMR (CDCl_3_, 500 MHz) d: 3.08 (s, 3H, Het-Me), 4.68 (s, 2H, SCH_2_), 7.10 (d, *J* = 7.8 Hz, 2H, ArH), 7.52 (s, 1H, thiazole-H), 7.61 (t, *J* = 8.0 Hz, 2H, ArH), 7.70 (t, *J* = 7.5 Hz, 1H, ArH). ^13^C-NMR (CDCl_3_, 125 MHz) d: 14.8, 28.3, 127.8, 130.8, 131.7, 131.9, 133.3, 136.4, 140.9, 148.2, 152.3, 152.9, 160.1. MS (ESI), *m/z*: 408 (M+1)^+^. Elemental anal. (%), calculated: C, 44.27; H, 2.72; N, 20.65; found: C, 44.44; H, 2.81; N,20.77.

*5-(5-((3,4-Dichlorobenzyl)thio)-4-phenyl-4H-1,2,4-triazol-3-yl)-4-methyl-1,2,3-thiadiazole* (**7n**). White crystals, yield 81%, m.p. 109–110 °C; ^1^H-NMR (CDCl_3_, 500 MHz) d: 3.06 (s, 3H, Het-Me), 4.49 (s, 2H, SCH_2_), 7.14 (d, *J* = 7.7 Hz, 2H, ArH), 7.30 (s, 1H, ArH), 7.44 (d, *J* = 8.3 Hz, 2H, ArH), 7.57 (t, *J* = 8.0 Hz, 2H, ArH), 7.66 (d, *J* = 7.5 Hz, 1H, ArH). MS (ESI), *m/z*: 423 (M+1)^+^. Elemental anal. (%), calculated: C, 49.77; H, 3.02; N, 16.12; found: C, 49.58; H, 3.35; N,16.01.

*3-(((4-Cyclopropyl-5-(4-methyl-1,2,3-thiadiazol-5-yl)-4H-1,2,4-triazol-3-yl)thio) methyl) benzonitrile* (**7o**). White solid, yield 82%, m.p. 133–134 °C; ^1^H-NMR (CDCl3, 400 MHz) δ: 0.79–0.83 (m, 2H, cyclopropane-CH_2_), 1.13–1.18 (m, 2H, cyclopropane-CH_2_), 2.95 (s, 3H, CH_3_), 2.97–3.02 (m, 1H, cyclopropane-CH), 4.53 (s, 2H, CH_2_), 7.37 (t, *J* = 7.22 Hz, 1H, Ph-H), 7.51 (d, *J* = 7.22 Hz, 1H, Ph-H), 7.71 (t, *J* = 7.84 Hz, 2H, Ph-H) ; MS (ESI), *m/z*: 355 (M+1)^+^; Elemental analysis for C_16_H_14_N_6_S_2_: found C 54.21, H 3.70, N 23.99; calc. C, 54.22; H, 3.98; N, 23.71.

*5-(4-Cyclopropyl-5-(prop-2-yn-1-ylthio)-4H-1,2,4-triazol-3-yl)-4-methyl-1,2,3-thiadiazole* (**7p**). White solid, yield 77%, m.p. 107–108 °C; ^1^H NMR (CDCl_3_, 400 MHz) δ: 0.78–0.94 (m, 2H, cyclopropane-CH_2_), 1.17–1.21 (m, 2H, cyclopropane-CH_2_), 2.24 (s, 1H, CH), 2.95 (s, 3H, CH_3_), 2.99-3.11 (m, 1H, cyclopropane-CH), 4.09 (s, 2H, CH_2_).MS (ESI): *m/z* 278 (M+1)^+^; Elemental analysis for C_11_H_11_N_5_S_2_: found C 47.88, H 4.23, N 25.14; calc. C, 47.63; H, 4.00; N, 25.25.

*5-(4-Cyclopropyl-5-(octylthio)-4H-1,2,4-triazol-3-yl)-4-methyl-1,2,3-thiadiazole* (**7q**). White solid, yield 81%, m.p. 67–68 °C; ^1^H-NMR (CDCl_3_, 400 MHz) δ: 0.79–0.86 (m, 5H, cyclopropane-CH_2_ and CH_2_), 1.14–1.24(m, 11H, cyclopropane-CH_2_ and CH_2_), 1.36–1.44 (m, 2H, CH_2_), 1.74–1.81 (m, 2H, CH_2_), 2.95(s, 3H, CH_3_), 2.99–3.05(m, 1H, cyclopropane-CH), 3.33 (t, 3H, CH_3_); MS (ESI) *m/z*: 352 (M+1)^+^; Elemental analysis for C_16_H_25_N_5_S_2_: found C 54.86, H 7.24, N 20.20; calc. C, 54.67; H, 7.17; N, 19.92.

### 3.4. Fungicidal Activities

Fungicidal activity of compounds **7a**–**q** against *Pseudoperonospora cubensis(PC)*, *Corynespora cassiicola(CC)*, *Pseudomonas syringae* pv. *Lachrymans(PS)* were evaluated according to reference [11], and a potted plant test method was adopted. Germination was conducted by soaking cucumber seeds in water for 2 h at 50 °C and then keeping the seeds moist for 24 h at 28 °C in an incubator. When the radicles were 0.5 cm, the seeds were grown in plastic pots containing a 1:1 (*v/v*) mixture of vermiculite and peat. Cucumber plants used for inoculations were at the stage of two seed leaves. Tested compounds and commercial fungicides were sprayed with a hand spray on the surface of the seed leaves on a fine morning, at the standard concentration of 500 μg/mL, iprodione, validamycin and topsin-M were used as a control. After 2 h, inoculations of *Pseudoperonospora cubensis*, *Corynespora cassiicola* were carried out by spraying a conidial suspension, inoculation of *Pseudomonas syringae* pv. *Lachrymans* was carried out by spraying fungal suspension. The experiment was repeated 4 times. After inoculation, the plants were maintained at 18–30 °C [mean temperature of 24 °C and above 80% relative humidity (RH)]. The fungicidal activity were evaluated when the nontreated cucumber plant (blank) fully developed symptoms. The area of inoculated treated leaves covered by disease symptoms was assessed and compared to that of nontreated ones to determine the average disease index. The relative control efficacy of compounds compared to the blank assay was calculated via the following equation:

relative control efficacy (%) = *(CK−PT)/CK* × 100%

where CK is the average disease index during the blank assay and PT is the average disease index after treatment during testing.

## 4. Conclusions

In summary, a series of 1,2,4-triazole derivatives containing 1,2,3-thiadiazole rings were synthesized in good yields. The preliminary bioassays showed that some of the compounds had good fungicidal activity. The present findings provided a powerful complement to the SARs of fungicides, and warrant future investigation of the mechanism of action of these analogues.
